# Challenges and Solutions in Advanced Management of Diabetic Foot Infections: A Review of Recent Studies

**DOI:** 10.1155/jdr/6715157

**Published:** 2025-09-30

**Authors:** Somaye Rashki, Mohammad Hossein Haddadi, Zeynab Marzhoseyni, Mansoor Khaledi, Mina Yekani, Mahdi Shooraj, Foroogh Neamati

**Affiliations:** ^1^Department of Laboratory Sciences, Sirjan School of Medical Sciences, Sirjan, Iran; ^2^Clinical Microbiology Research Center, Ilam University of Medical Sciences, Ilam, Iran; ^3^Department of Paramedicine, Amol School of Paramedical Sciences, Mazandaran University of Medical Sciences, Sari, Iran; ^4^Department of Microbiology and Immunology, School of Medicine, Shahrekord University of Medical Sciences, Shahrekord, Iran; ^5^Pediatric Health Research Center, Tabriz University of Medical Sciences, Tabriz, Iran; ^6^BSc in Medical Laboratory Sciences, Mazandaran University of Medical Sciences, Sari, Iran; ^7^Infectious Diseases Research Center, Kashan University of Medical Sciences, Kashan, Iran

**Keywords:** diabetes, diabetic foot infections, diabetic wound, diagnosis, pathogenesis of diabetes

## Abstract

Diabetic foot infections (DFIs) represent a significant and prevalent complication of diabetes, contributing to considerable morbidity, mortality, and healthcare costs globally. These infections, ranging from mild monomicrobial cases to severe polymicrobial infections, often require hospitalization and can result in limb amputation. The microbial etiology of DFIs is diverse, with common pathogens including *Staphylococcus aureus* (*S. aureus*), *Escherichia coli*, *Pseudomonas aeruginosa*, and various anaerobes. The pathogenic mechanisms of DFIs are complex, involving peripheral neuropathy, vascular insufficiency, and immune dysfunction, all exacerbated by a hyperglycemic state. Despite advances in treatment, the increasing prevalence of antimicrobial resistance, particularly among methicillin-resistant *S. aureus* (MRSA) strains, presents a major challenge to managing these infections effectively. This review systematically examines the pathogenesis, diagnostic techniques, microbial profiles, and treatment strategies for DFIs, with an emphasis on antibiotic resistance and new therapeutic approaches. Furthermore, the article highlights the need for a multidisciplinary approach, including early diagnosis, appropriate antibiotic therapy, advanced wound care, and patient education to mitigate the risk of severe complications. Given the rising global burden of diabetes, improved management of DFIs remains critical for reducing the incidence of amputations and minimizing the economic burden on healthcare systems.

## 1. Introduction

Diabetes mellitus (DM) is a metabolic disorder characterized by chronic hyperglycemia, which can lead to serious complications, reduced quality of life, and increased mortality. With over 400 million people affected globally, DM poses a significant global health burden. Approximately 10% of these cases are Type 1 diabetes, while 90% are Type 2 [1].

A prevalent and serious complication of DM is the development of diabetic foot ulcers (DFUs), which affect approximately 6.3% of diabetic patients [2]. The progression of DFIs can result in tissue ulceration and gangrene, leading to conditions that may necessitate amputation if left untreated [3]. The presence or absence of infection, as well as its severity (e.g., cellulitis and osteomyelitis), is a critical factor in guiding clinicians toward the appropriate course of action [4]. The management of DFIs involves careful consideration of advanced diagnostic techniques and comprehensive antibiotic susceptibility profiling, especially in cases involving osteomyelitis [5].

The microbial profile of DFIs ranges from monomicrobial infections, predominantly involving *Staphylococcus aureus* (*S. aureus*), to polymicrobial infections that include Gram-positive cocci, Gram-negative bacilli, and anaerobes [6]. Polymicrobial infections are common, with frequently isolated pathogens including *Escherichia coli* (*E. coli*), *Proteus mirabilis* (*P. mirabilis*), *Pseudomonas* species, and *S. aureus*. The interaction among these microbes and their resistance to antimicrobials complicates treatment and often leads to poor outcomes [7]. Antibiotic resistance can result in extended infection durations, increased healthcare costs, and an elevated risk of severe outcomes, including limb amputation [8].

Effective DFI management requires a multidisciplinary approach that accounts for systemic complications such as peripheral neuropathy and impaired immune responses [6]. Thus, accurate assessment of infection severity, precise identification of the responsible pathogens, antimicrobial resistance profiling, and the implementation of targeted therapeutic strategies are essential for treatment and for reducing the risk of severe complications, such as limb amputation and death [5].

This review is aimed at providing a comprehensive evaluation of DFIs, covering their pathogenesis, epidemiology, diagnostic and therapeutic strategies, and prevention methods. The objective is to identify the existing challenges and explore opportunities for improving clinical outcomes.

## 2. Pathogenesis and Risk Factors

DFUs are full-thickness dermal wounds typically found on weight-bearing or trauma-prone areas of the foot. Their pathogenesis involves neuropathy, vascular insufficiency, and immune dysfunction, all of which are exacerbated by chronic hyperglycemia [9]. In the next paragraph, the mechanisms involved in the DFU pathogenesis are reviewed.

Peripheral neuropathy, one of the most common complications of diabetes, affects over 60% of diabetic patients and significantly increases the risk of foot trauma and ulceration [10]. Several impaired physiological pathways are linked to peripheral nerve injury, such as ATP deficiency, which affects neurotransmitter or cotransmitter release at many autonomic nervous system (ANS)–effector synapses, promotes axonal degeneration, particularly in mitochondria-rich axons, and causes delayed diabetic neuropathy. Peripheral nerve injury is frequently caused by the accumulation of sorbitol and fructose. In diabetic conditions, high glucose levels enhance the affinity of aldose reductase (AR) for glucose, resulting in increased sorbitol synthesis [11]. One of the primary effects of neuropathy is denervation, which initiates foot muscle atrophy. It is associated with anatomical changes in the foot and muscle paralysis [10].

The accumulation of sorbitol impairs Na+/K+-ATPase activity and slows nerve conduction [10, 11]. Moreover, the depletion of nicotinamide adenine dinucleotide phosphate (NADPH), which is required for the synthesis of antioxidant molecules such as glutathione, leads to increased oxidative stress in diabetic patients [11].

Protein kinase C (PKC) activation under hyperglycemic conditions exacerbates vascular dysfunction and contributes to microvascular complications [12, 13]. Autonomic neuropathy impairs sweat gland function, leading to dry skin and cracks that increase susceptibility to microbial invasion [14, 15]. In addition, impaired angiogenesis, marked by reduced VEGF and FGF-2 expression, and elevated thromboxane A2 levels lead to vasoconstriction, impaired wound healing, and increased ulceration risk [14–17]. These findings highlight the importance of addressing angiogenic impairments to improve wound healing outcomes in diabetic patients [18].

Wound healing begins with hemostasis and progresses through inflammation, proliferation, re-epithelialization, granulation, and finally remodeling [18, 19]. Neutrophils play an important role in wound healing through neutrophil extracellular traps (NETosis), an immune response that occurs via the release of granular molecules to eliminate foreign pathogens [20, 21]. Diabetic patients with dysregulated NETosis show increased upregulation of proinflammatory cytokines and production of free radicals and superoxide, which impairs proliferation and re-epithelialization [18, 22].

Hyperglycemia also promotes the formation of advanced glycation end-products (AGEs), which bind to AGE receptors (RAGE) and trigger NF-*κ*B-mediated inflammatory cascades [23]. Subsequently, the cell becomes susceptible to lysis due to the continuous release of inflammatory cytokines and apoptosis [24]. Collagen degradation by matrix metalloproteinases (MMPs) contributes to extracellular matrix disorganization and ulcer persistence. However, MMPs are potential therapeutic targets to accelerate DFU healing [25].

DFUs arise from a combination of personal and clinical factors, as well as external conditions. Risk factors for DFUs include age, gender, smoking, poor glycemic control, neuropathy, peripheral artery disease (PAD), and foot deformities [26, 27]. Reduced sensory perception predisposes individuals to complications such as callus formation, ulceration, and a susceptibility to infections. In immunocompromised patients, the presence of these factors can significantly delay wound healing, thereby increasing their vulnerability to infection [28].

PAD is a condition that partially or completely occludes the noncardiac and nonintracranial arteries. Consequently, tissue ischemia and insufficient blood supply occur when peripheral arteries of the upper and lower limbs are damaged [29]. PAD is associated with chronic ischemic ulcers, susceptibility to infection, and the need for amputation [30]. The higher frequency of foot amputations in diabetic patients suffering from PAD, compared to those without PAD, indicates the strong association between PAD and DFU [31].

According to the Task Force of the Foot Care Interest Group of the American Diabetes Association, foot deformity is the third most common risk factor for DFU [32]. Deformities of the metatarsophalangeal joints (MTPJs), such as hammer and claw toes and hallux valgus, are frequently observed in diabetic patients [33]. Repetitive ischemia–reperfusion cycles (RIRCs) stimulate inflammatory cytokine production and tissue injury. It is likely that foot deformities, metatarsal head pressure, and ulcer formation are due to RIRCs [34]. Initially, prolonged elevation of plantar pressure causes callus thickening and hyperkeratosis, leading to ulcer formation [35]. Pathological alterations in tendons, including a thickened Achilles tendon and irregular tendon structure, restrict joint mobility and impair ankle dorsiflexion, increasing the risk of ulcer formation [33].

**Figure 1:**
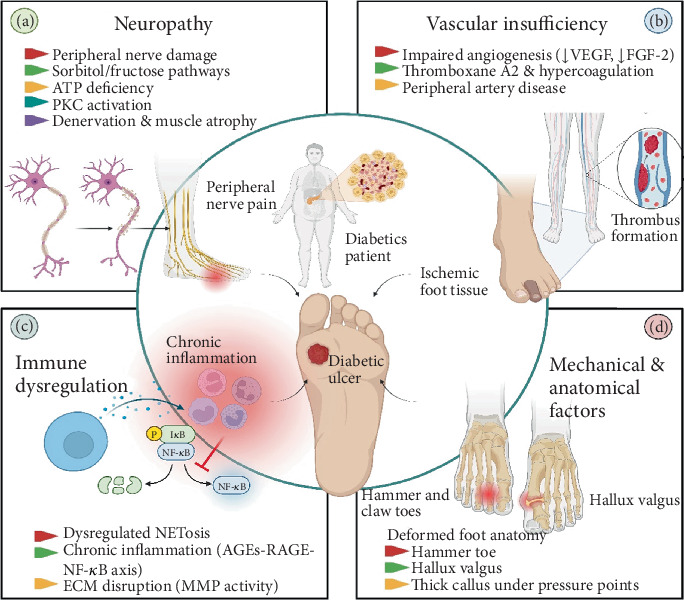
DFU development and progression. The central illustration shows a diabetic patient with a foot active ulcer, representing the clinical outcome of multiple intertwined pathologies. Five major categories radiate outward including neuropathy (a), vascular insufficiency (b), immune dysregulation (c), and mechanical and anatomical factors (d).

Although immunological responses are sometimes more damaging than the triggering agents, dysfunctional immune responses often permit bacterial infection and related inflammation, which presumably arise from bacterial pathogenesis mechanisms leading to ulcer formation [35]. Clayton et al. revealed that lifestyle and behavioral factors, including smoking, sedentary behavior, and obesity, were risk factors for DFUs [18, 36]. A schematic overview of the pathophysiological mechanisms and risk factors contributing to the development and progression of DFUs is illustrated in Figure 1.

## 3. Epidemiology

Epidemiological data indicate that approximately 15%–20% of diabetic patients develop foot ulcers, and a high percentage (20%–60%) of these ulcers are at risk of becoming infected [2]. Studies of DFUs suggest that the risk of developing an infection increases with the duration of diabetes and the presence of peripheral neuropathy, PAD, and other common comorbidities in diabetic patients [37]. In these patients, DFI severity is associated with the duration of hospitalization and the incidence of amputation [38]. In regions with limited healthcare resources and a high incidence of DFIs, hospitalization and amputations are more common due to inadequate access to preventive care and treatment facilities. The rising global prevalence of diabetes, particularly in low- and middle-income nations, is predicted to increase the burden of DFIs correspondingly [39].

The economic burden of DFIs is substantial. In the United States, each episode of care may cost between $9000 and $30,000, contributing to an annual burden exceeding $5 billion [40]. The economic impact of DFIs is profound, encompassing both direct medical costs, such as hospital admissions, surgical interventions, antibiotic therapy, and outpatient visits, and indirect expenses like loss of productivity, long-term disability, and the need for social care [41]. These costs highlight the critical need for effective prevention and management strategies to reduce the incidence of DFIs and the consequent financial burden. Implementing comprehensive diabetic foot care programs, which include regular screening, patient education, and timely intervention, can substantially reduce the prevalence of foot ulcers and infections, thereby lowering the overall healthcare costs associated with DFIs [42].

Based on the Infectious Diseases Society of America (IDSA) guidelines, DFIs are typically classified as mild, moderate, or severe based on infection depth, tissue involvement, and systemic symptoms [43]. Mild infections are confined to the skin and subcutaneous tissues without systemic symptoms [44]. Moderate infections involve deeper tissues and might exhibit signs of systemic infection but are not limb- or life-threatening [45]. Severe infections are defined by extensive deep tissue involvement, systemic signs of infection, or the need for amputation [46]. This classification informs clinical decisions regarding hospitalization, surgery, and antibiotic therapy.

## 4. Etiologic Agents

The microbial etiology of DFIs is diverse. DFIs are typically polymicrobial diseases caused by more than one type of microbe that are present simultaneously and interacting to cause a specific pathology such as DFI (Table 1). Aerobic Gram-positive cocci, mainly *S. aureus*, are the most frequently isolated pathogens, with a significant proportion being MRSA strains [66]. Gram-negative bacteria, including *Pseudomonas aeruginosa* (*P. aeruginosa*) and numerous *Enterobacteriaceae*, are typically involved, especially in chronic or previously treated infections [67]. Chronic infections often involve Gram-negative bacteria like *P. aeruginosa* and *E. coli*, with anaerobes contributing to necrotic or deep wounds. Fungal pathogens may also complicate treatment [50].

Local antibiotic resistance patterns, which can vary significantly between regions and healthcare settings, should ideally guide treatment [68]. In acute DFIs, empirical antibiotic therapy is often initiated to mitigate the risk of progressive tissue damage like gangrene or systemic infection, as diagnostic delays may compromise outcomes. Tailored regimens are subsequently adjusted based on culture results and clinical response. Antimicrobial stewardship and pathogen-specific therapy are critical [69].

On the other hand, misdiagnosis of DFI can lead to excessive and improper use of antibiotics. In addition, the prevalence of resistant pathogens is increasing due to the extensive use of broad-spectrum antibacterial agents, biofilm formation, and the expression of resistance genes. Past antibiotic use may have affected the bacterial spectrum of DFI. Accordingly, the causative organisms of DFI are diverse and influenced by lifestyle, location, environment, economy, and awareness. Therefore, the key consideration in DFI treatment is selecting appropriate antibiotics [70].

## 5. Diagnosis Methods

The early detection of infection is crucial to prevent the progression of DFIs into deeper tissues and to avoid complications such as osteomyelitis or amputation. To accurately diagnose DFI, it is important to evaluate several factors and combine all the information available. A comprehensive diagnostic approach should integrate multiple assessment tools rather than rely on a single criterion [71].

### 5.1. Microbiological Sampling

Obtaining a proper sample is essential for an accurate diagnosis. Common methods include swabbing the wound surface, tissue biopsy, or aspiration of purulent secretions [72]. Generally, tissue biopsy is the gold standard, and culturing debrided infected tissue is an important method for detecting the etiological agents [1]. Diagnosis of osteomyelitis relies on surgical bone sampling and microbiological methods. For osteomyelitis diagnosis, a surgically obtained bone sample (obtained surgically or via transcutaneous biopsy) is often required depending on clinical circumstances, including whether surgical intervention is otherwise indicated [73]. In the past, traditional culture methods and phenotypic identification were used for DFI diagnosis. But today, molecular assays such as the 16S rDNA PCR amplification and sequencing have been developed [72]. However, molecular assays like 16S rDNA PCR and sequencing are not recommended as first-choice diagnostics by the IWGDF/IDSA due to high costs, limited availability, and lack of demonstrated clinical benefit over conventional methods [74].

### 5.2. Biochemical Markers

Many studies have reported that clinical markers can be important indicators in DFI detection. In this regard, inflammatory markers like erythrocyte sedimentation rate (ESR), C-reactive protein (CRP), procalcitonin (PCT), and white cell count (WCC) are useful in differentiating infected from noninfected DFUs and diabetic foot osteomyelitis (DFO) [75].

### 5.3. Radiological Diagnosis

Radiographic imaging is an ideal technique for diagnosing osteomyelitis due to its detailed anatomical display, high sensitivity, and ability to detect infection early [76]. Magnetic resonance imaging (MRI) is particularly useful for revealing early bone and soft tissue changes. MRI can detect bone marrow edema, soft tissue inflammation, abscesses, and joint effusions [76].

### 5.4. Clinical Diagnosis

Clinical diagnosis is the primary method for identifying this infection, relying on medical signs and patient-reported symptoms. DFIs are diagnosed per IWGDF/IDSA criteria [74], including purulent discharge, erythema, warmth, pain, or systemic inflammation. Additional clinical observations, such as odor and friable tissue, may support but are not definitive for infection. In a diabetic foot, signs of superficial infections like erythema, warmth, and edema may be less clear. Typically, systemic symptoms such as fever, chills, and swollen lymph nodes are absent [77]. Thus, clinical assessment remains essential for accurate diagnosis, as systemic indicators are often lacking.

## 6. Treatment

The effective management of DFI requires proper antibiotic treatment, surgical drainage, removal of dead tissues, proper wound care, correction of metabolic problems, and revascularization when needed [78].

### 6.1. Photodynamic and Ozone Therapy

Photodynamic therapy (PDT) utilizes light-activated photosensitizers to generate reactive oxygen species (ROS) that kill microbes and stimulate healing. PDT is an adjunct treatment for DFI because of its unique properties, such as reducing the microbial load and inflammation; enhancing healing; and stimulating the proliferation of fibroblasts, collagen, and elastin to accelerate wound healing [79].

On the other hand, ozone therapy improves the activity of antioxidant enzymes, such as superoxide dismutase and oxidized glutathione reductase, while also inhibiting biofilm formation on epithelial cells and exhibiting antibacterial effects [80]. Additionally, it reduces proinflammatory cytokines, like IL-6 and TNF-*α*, promotes wound healing, and mitigates bacterial colonization damage. These combined antibacterial, anti-inflammatory, and antioxidant properties make ozone therapy a compelling option for DFI management [80].

### 6.2. Biologic Compounds

Biologic therapies derived from human, plant, or microbial sources offer promising adjuncts in DFI treatment. Biologic compounds that can be used for DFI treatment are summarized in Table 2. Numerous studies have indicated that platelets may serve as an adjunct to improve the process of wound healing [85]. Platelet-rich plasma (PRP) accelerates wound healing by promoting cell proliferation and exhibits antibacterial activity against pathogens like *E. coli* and *S. aureus* [86]. One of the biological compounds that can be used in treatments for various chronic skin conditions is honey, which exhibits antibacterial, antioxidant, anti-inflammatory, and biocompatible activities [87]. All these characteristics suggest that honey could be a promising candidate for further investigation in efficient pharmaceutical applications, including DFI treatment; however, well-designed comparative studies are needed to validate its efficacy and industrial feasibility [87]. Moreover, in recent years, studies have reported that antimicrobial peptides (AMPs) play important roles in DFI treatment. The mechanism by which AMPs exert their therapeutic potential includes stimulating cytokine production and promoting keratinocyte migration, proliferation, and angiogenesis. In this respect, AMPs such as LL-37, CW49, Pexiganan, and hBD-2 have potential applications in DFI treatment [83].

### 6.3. Antibiotic Therapy

Antibiotic therapy should be guided by infection severity, prior treatment, culture results, and patient-specific factors such as allergies [69]. In many temperate regions, the most significant microorganisms causing DFI are often *S. aureus*; however, in warm climates, Gram-negative bacilli may predominate [88, 89]. Treatment failures often occur due to inadequate penetration, the presence of biofilms, or unaddressed abscesses. Timely culture and sensitivity testing can improve therapeutic efficacy [69].

### 6.4. Surgical Treatment

Surgery is often necessary for advanced DFUs to drain abscesses, remove necrotic tissue, or correct anatomical deformities. The goal is to halt the spread of infection and prevent amputation. Surgical interventions can range from minor debridement to extensive procedures, depending on the severity of infection [90].

### 6.5. Wound Dressings

**Figure 2:**
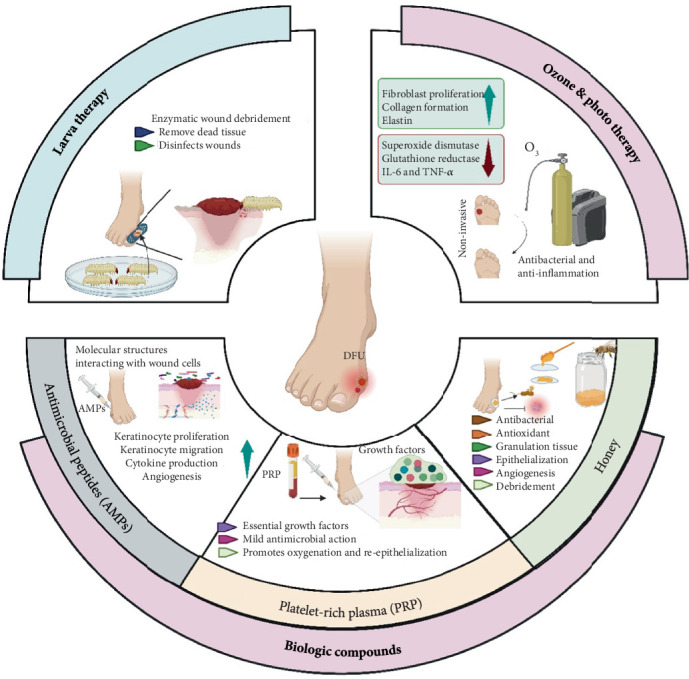
Advanced therapies for DFU.

Both natural and synthetic materials can be used to create wound dressings. The pharmaceutical industry has facilitated the extensive use of these materials due to their special physicochemical characteristics, such as biodegradability, biocompatibility, and minimal antigenicity. Various factors should be considered when choosing the appropriate dressing for a diabetic foot. As depicted in Figure 2, an appropriate dressing should have a number of critical characteristics, including high levels of biocompatibility and biodegradability. Thus, a more efficient dressing creates a warm and moisturized environment that enhances tissue regeneration; allows gas exchange; and promotes cell migration, proliferation, and neovascularization [91]. There are several types of dressings, such as films, hydrogels, acrylics, hydrocolloids, calcium alginates, hydrofibers, and foams [92].  Table 3 presents an overview of dressings and their practical use in DFI treatment.

### 6.6. Larval Therapy

Larval therapy, or maggot therapy, was first introduced in the United States in the 1940s but is still in its infancy. Several clinical and preclinical studies have demonstrated that larval therapy can be effective in the treatment of DFI through direct contact. The saliva of maggots contains enzymes that enhance bacterial elimination, accelerate disinfection and debridement, and improve wound healing. In addition, maggots produce AMPs that preferentially reduce bacterial load, particularly in antibiotic-resistant infections [98].

## 7. Management and Prevention

DFIs are intricate and multifaceted medical conditions that require a comprehensive approach for their diagnosis, treatment, and prevention. Effective prevention strategies are crucial to reducing the incidence and recurrence of DFIs [99]. Strict glycemic control is fundamental to preventing diabetic complications, including diabetic retinopathy, nephropathy, and neuropathy in individuals with insulin-dependent DM [100]. However, intensive glucose regulation must be balanced against the risk of hypoglycemia [101].

There is substantial evidence indicating that adopting healthy lifestyle habits, such as a nutritious diet, moderate weight loss, and regular physical activity, can effectively maintain normal blood sugar levels and reduce the risk of complications related to diabetes. Furthermore, these lifestyle changes play an important role in managing hyperglycemia and maintaining optimal blood sugar levels [102]. Engaging in aerobic exercise or walking can significantly improve physical health and help regulate blood sugar levels in patients with DFUs [103].

Another effective method for preventing complications such as DFIs is to educate patients, raise awareness of diabetes-related complications, and emphasize the importance of developing a foot care plan. Individuals with diabetes need to be informed about critical aspects of managing their condition [104]. To protect their feet, it is important that at-risk diabetic patients avoid walking barefoot, wearing only socks, or using thin-soled standard slippers, whether indoors or outdoors [105]. It is also essential to educate diabetic individuals and their family members about the importance of using suitable footwear to prevent DFUs.

Additionally, individuals at intermediate or high risk of DFUs should be advised to obtain footwear from a trained professional to ensure that the shoes fit properly, protect the feet, and conform to their shape to prevent complications [106]. Performing daily foot inspections, including checking the areas between the toes, is essential for diabetic patients. This practice helps in the early detection of any signs of infection or injury. Furthermore, toenails should be trimmed straight across to avoid the development of ingrown toenails, which can lead to infections [107]. Besides, monitoring foot temperature at home is a self-management intervention highlighted in international guidelines to help reduce the risk of foot ulceration in diabetic individuals. If a localized increase in foot temperature is detected, patients should reduce their ambulatory activity [108]. Proper foot hygiene, including careful washing and drying, is crucial for diabetic individuals to prevent infection and other complications [109]. Research reported that utilizing an innovative intelligent insole system, which provides continuous plantar pressure feedback and promotes offloading, can significantly decrease the occurrence of recurrent DFUs over an 18-month period in high-risk diabetic patients. This system assists patients in monitoring pressure on their feet, thus preventing the formation of ulcers [110].

Moreover, studies estimated that cigarette smoking alone could directly contribute to at least 25 million cases of Type 2 diabetes mellitus (T2DM) worldwide. Although individuals who quit smoking within the past 5 years still faced an elevated risk of developing T2DM, this risk gradually decreased over time. After 10 years of cessation, the risk level for former smokers became comparable to that of individuals who had never smoked [111]. These findings emphasize the critical role of smoking cessation in lowering the risk of severe complications in individuals with diabetes [112]. Implementing preventive strategies for diabetes patients can significantly alleviate the global patient and economic burden of diabetic foot disease. By minimizing the risk of ulceration, the likelihood of infection, hospitalization, and lower extremity amputation in these individuals is reduced [113].

## 8. Conclusion

Diabetic foot infections remain a major global health concern, causing substantial morbidity, mortality, and economic burden. Despite advancements in diagnostics and therapeutics, challenges persist due to the multifactorial pathogenesis of DFIs, the emergence of multidrug-resistant organisms, and the complexity of patient management. A comprehensive, evidence-based, and multidisciplinary approach is essential to improve clinical outcomes and reduce amputation rates. Accordingly, regular foot screening, patient education, and prompt, appropriate treatment of infections are critical for decreasing the burden of DFIs.

Despite considerable progress in diabetic foot care, critical clinical challenges remain, particularly in the early detection of complications, ensuring patient adherence, and effectively integrating technology into prevention and management strategies for DFIs. A key limitation lies in the underutilization of personalized educational interventions. Additionally, innovations such as AMPs and PRP remain insufficiently implemented. It is recommended that multidisciplinary teams be established to coordinate individualized treatment plans, including surgical, pharmaceutical, and regenerative options. Emerging evidence demonstrates that MDTs reduce amputation rates, improve healing, and enhance long-term survival [114].

Even though current clinical guidelines emphasize the importance of foot hygiene, suitable footwear, and lifestyle changes, many patients continue to be inadequately informed or struggle to follow preventive recommendations.

Future research should prioritize the development and evaluation of adaptive AI-enabled platforms that combine patient education, remote monitoring tools (such as thermography and pressure sensors), and real-time clinical feedback to support proactive management of DFIs. A proactive, integrated, and patient-centered strategy is vital to reduce the burden of diabetic foot infections. Addressing current diagnostic and therapeutic limitations, promoting preventive care, and fostering global collaboration are key to improving patient outcomes and healthcare sustainability.

## Figures and Tables

**Table 1:** Common microbes in diabetic foot infections and their key characteristics.

**Organisms**	**Pathogenesis**	**Antibiotic resistance**	**Typical sources of infection**	**Impact on diabetic ulcer**	**Ref**
*S. aureus* (including MRSA)	Severe	Resistant (especially MRSA)	Skin infections	Significant	[47]
*Streptococcus* spp.	Mild to severe	Varies	Superficial to deep tissue infections	Aggravating factor	[48]
*P. aeruginosa*	Severe	Highly resistant	Chronic wounds	Significant	[49]
*E. coli*	Moderate to severe	Varies	Urinary tract infections, wound infections	Associated with fecal contamination in wounds	[50]
*Enterococcus* spp.	Opportunistic	Resistant (often)	Normal intestinal flora, wound infections	Aggravating factor	[51]
*P. mirabilis*	Moderate	Varies	Wound infections	Causes foul-smelling wounds	[52]
*K. pneumoniae*	Severe	Resistant	Severe infections	Aggravating factor	[53]
*Enterobacter* spp.	Opportunistic	Possibility of resistance	Healthcare-associated infections	Aggravating factor	[54]
*A. baumannii*	Severe	Highly resistant	Hospital settings	Significant in patients with prolonged stays	[55]
*Serratia marcescens*	Moderate	Possibility of resistance	Nosocomial infections	Aggravating factor	[56]
*Morganella morganii*	Severe	Varies	Severe infections	Aggravating factor	[57]
*Citrobacter freundii*	Moderate	Possibility of resistance	Systemic and localized infections	Aggravating factor	[58]
*Coagulase-negative Staphylococci*	Mild	Varies	Device-related infections	Important in biofilm-related infections	[59]
*Corynebacterium* spp.	Mild	Typically nonresistant	Skin infections	Significant in immunocompromised individuals	[60]
*Anaerobes*	Severe	Varies	Polymicrobial infections	Significant	[61]
*Enterococcus faecalis* and *faecium*	Opportunistic	Resistant (often)	Gut-derived infections	Aggravating factor	[62]
*Stenotrophomonas maltophilia*	Emerging pathogen	Highly resistant	Nosocomial infections	Significant in hospital settings	[63]
*Mycobacterium* spp.	Mild to moderate	Varies	Chronic infections	Significant in immunocompromised individuals	[64]
Fungal species (e.g., *Candida*)	Mild to severe	Varies	Colonization and infection	Aggravating factor	[65]

**Table 2:** Biological agents that can be used to treat DFIs.

**Biological agents**	**Function**	**Administration**	**Ref**
Growth factors	Formation of granulation tissue, prevention of amputation in patients, reduction of ulcer size	Local	[81]
Neuropeptides	Activation of growth factors	Local, systemic	[82]
Antimicrobial peptides	Regulate various processes of inflammation and wound closure, antiviral, antifungal, antibacterial, antitumor activities	Local, systemic	[83]
Honey	Antibiotic, antioxidant, and anti-inflammatory Contraction of wounds	Local	[74]
Plant extracts	Anti-inflammatory and antimicrobial properties Triggering diverse growth factors, cytokines, and chemokines, tissue regeneration	Local	[84]

**Table 3:** Dressing types and their practices in DFI treatment.

**Types of dressings**	**Function**	**Type of wounds**	**Ref**
Hydrogels	The exchanging of nutrients and oxygen between the wound bed and the surrounding tissues, reduction of inflammation, reduce bacterial growth, promoting angiogenesis	Dry to mildly exudating wounds, burns	[93]
Alginate dressings	Exceptional absorbent, hemostatic, antibacterial activity	Moderate to heavily exudating wounds	[94]
Poly (acrylic acid)	Antibacterial activity, inhibition of proteases, collagen deposition, re-epithelialization, granulation, and vascularization, destroying the cell membranes of bacteria	Chronic wounds, diabetic foot ulcers (DFUs), infected wounds, wounds with biofilm	[95]
Hydrocolloids	Wound moisture maintenance, promoting wound healing by absorbing exudate, promoting a rapid regrowth of skin over the wound bed	Light to moderately exudating wounds	[96]
Hydrofibers	Healing enhancement, pain reduction, scarring reduction, bacterial growth inhibition, conformability of the dressing with the wound bed, dead space reduction	Moderate to heavily exudating wounds, deep wounds, surgical wounds, infected wounds	[97]

## Data Availability

The authors confirm that the data supporting the findings of this study are available within the article.
